# Isotopic evidence for dietary niche overlap between barking deer and four-horned antelope in Nepal

**DOI:** 10.1186/s40709-015-0029-0

**Published:** 2015-05-06

**Authors:** Krishna Prasad Pokharel, Elizabeth Yohannes, Ioanna Salvarina, Ilse Storch

**Affiliations:** Wildlife Ecology and Management, Faculty of Environment and Natural Resources, University of Freiburg, Freiburg, Germany; Limnological Institute, University of Konstanz, Konstanz, Germany

**Keywords:** Herbivores, Niche, Resource management, Resource partitioning, Seasonal diet, Stable isotopes

## Abstract

**Background:**

Morphologically similar sympatric species may have a high degree of niche overlap. Barking deer *Muntiacus vaginalis* and four-horned antelope *Tetracerus quadricornis* are solitary ungulates of the Indian sub-continent. Limited information is available regarding their trophic ecology, particularly of the endemic four-horned antelope. We present stable carbon (δ^13^C), nitrogen (δ^15^N), and sulphur (δ^34^S) isotopic values, and nitrogen content (%N) of faeces from barking deer and four-horned antelope living in lowland Nepal to assess trophic niche differentiation of these herbivores along the browser-grazer continuum. We also describe trophic differences between those two species in ecological niches and seasonal effects on their diets.

**Results:**

We found that the barking deer and four-horned antelope consumed C_3_ plant sources exclusively. The niche partitioning in their diet was reflected by δ^34^S values. Some seasonal effects observed were: δ^13^C and δ^15^N were significantly lower in the dry season diet of four-horned antelope than that of barking deer, while δ^34^S values were significantly higher in the winter diet; monsoon diet was similar for both species. Faecal N levels for barking deer and four-horned antelope were similar throughout all the seasons, indicating that both species adapted their feeding behaviour so as to maximize protein intake, in accordance with season and environment.

**Conclusions:**

Barking deer and four-horned antelope both are browsers; their dietary sources overlapped during monsoon but differed during the dry season. Conservation actions focused on resource management during the dry season to reduce food scarcity and competition over limited resources is likely to be the most effective.

**Electronic supplementary material:**

The online version of this article (doi:10.1186/s40709-015-0029-0) contains supplementary material, which is available to authorized users.

## Background

The ecological niche of a species is a combination of the biotic and abiotic factors that affect the fitness (successful reproduction) of an individual or a population of that species [1,2]. According to niche theory, ‘coexisting species should differ in their ecological requirements by at least some minimal amount to avoid competitive exclusion’ ([3], pp. 2141). Niche partitioning among sympatric herbivores are largely related to the differences in body size [4,5], and in part supported by predation [6]. Thus, morphologically similar herbivores may have high levels of ecological similarities that could result in competition when population density is high and resources are limited [6]. Hence, potentially competing sympatric species tend to partition their niches to avoid or lessen competition [3,6-8]. The mechanism allowing for niche partition occurs along at least three niche axes: spatial, trophic, and temporal [9]. The trophic niche is a major niche factor frequently partitioned [6,10]. Furthermore, trophic niche interactions between sympatric species can provide information on potential competition. However, Hubbell’s neutral theory of biodiversity has challenged the niche theory [11]; he states that coexistence of species is possible without niche partitioning [12]. Furthermore, relationships between trophic niche overlap and competition are also an issue of controversy [13,14].

The trophic niche of ungulates is often classified along a browser/grazer continuum [4,15,16]. This rather coarse classification does not sufficiently reflect dietary differences of coexisting species [9,17]. Stable isotope analysis of animals’ faeces has been widely used as a reliable source of information for dietary signatures over a range of temporal and spatial scales. Faeces retain isotopic dietary information of several hours to days, thus, isotope analysis of faeces presents an attractive non-invasive tool to assess mammals’ short-term dietary patterns [15,18,19]. The stable carbon isotope (δ^13^C) in faeces reliably reflects the proportion of C_3_ plants (browse) to C_4_ plants (grass) ingested by the consumer [20-22]. The stable nitrogen isotope ratio (δ^15^N) and faecal nitrogen content (%N) both provide information on the trophic level of an organism, and indicate physiological stress and nitrogen uptake levels [23-25]. The stable sulphur isotope ratio (δ^34^S) indicates the primary sulphur source in foods [26,27]. Hence, changes in δ^13^C, δ^15^N and δ^34^S values in the diet of herbivores may reflect a change in trophic niche and foraging habitat.

Most of the studies on trophic niche interactions among sympatric herbivores have been carried out in temperate zones [28-30] and tropical Africa [17,31,32]. However, few studies have focused on the diets of sympatric herbivores in subtropical Asia [33-35]. Moreover, those studies largely focused on sympatric ungulates with different body size. Therefore, barking deer *Muntiacus vaginalis* (Boddaert, 1785; BD hereafter) and four-horned antelope *Tetracerus quadricornis* (de Blainville, 1816; FHA hereafter), which are solitary herbivores with similar morphologies (shoulder height 55–65 cm, body mass 18-21 kg; [36-38]) provide a good opportunity to study the trophic niche interactions between sympatric herbivores.

Overall, BD are described as exhibiting a wide variety of feeding habits that range from selective feeder [39,40] to grazer [41] and mixed feeder [42], while FHA are defined as browsers and mixed feeders [38,43,44]. So far, most authors have reported qualitative descriptions, and quantitative assessment of the dietary sources of these two species is still lacking. Little is known about the dietary sources particularly that of FHA in Nepal, and whether there is a trophic niche partitioning between the two species. To fill these gaps, we assessed elemental stable isotope analysis (δ^13^C, δ^15^N, δ^34^S), and %N values from faeces to assess seasonal diet variation for sympatric BD and FHA in Bardia National Park, Nepal. Based on predictions from niche theory that ‘coexisting species should differ in their ecological requirements by at least some minimal amount to avoid competitive exclusion’ [3], we discuss the partitioning of dietary sources in terms of stable isotopes of these sympatric species. We expected to find the most pronounced differences in dietary sources and faecal isotopic values in seasons with limited food abundance.

## Results and discussion

### Faecal stable isotopes and dietary sources

According to the MANOVA, stable isotopes of faeces of the BD and FHA did not differ in terms of δ^13^C, δ^15^N, δ^34^S and %N values (Pillai’s Trace: F_4,47_ = 1.546, *p* = 0.204), and sampling sites had no effect on diets of the two species (Pillai’s Trace: F_4,47_ = 1.862, *p* = 0.133). Furthermore, there was no effect of combined interactions between species and sampling sites (species × sampling sites) (MANOVA; Pillai’s Trace: F_8,100_ = 1.38, *p* = 0.22). However, there was a seasonal effect (Pillai’s Trace: F_4,47_ = 10.040, *p* < 0.01) as well as combined effect of interactions between species and seasons (species × seasons) (MANOVA; Pillai’s Trace: F_8,100_ = 2.61, *p* = 0.012). One-way ANOVA also revealed that stable isotopes of BD and FHA were similar in terms of δ^13^C, δ^15^N and %N values (Additional file 1). However, δ^34^S values were higher for FHA faeces than for BD faeces (F = 4.60, *p* = 0.04).

**Figure 1:**
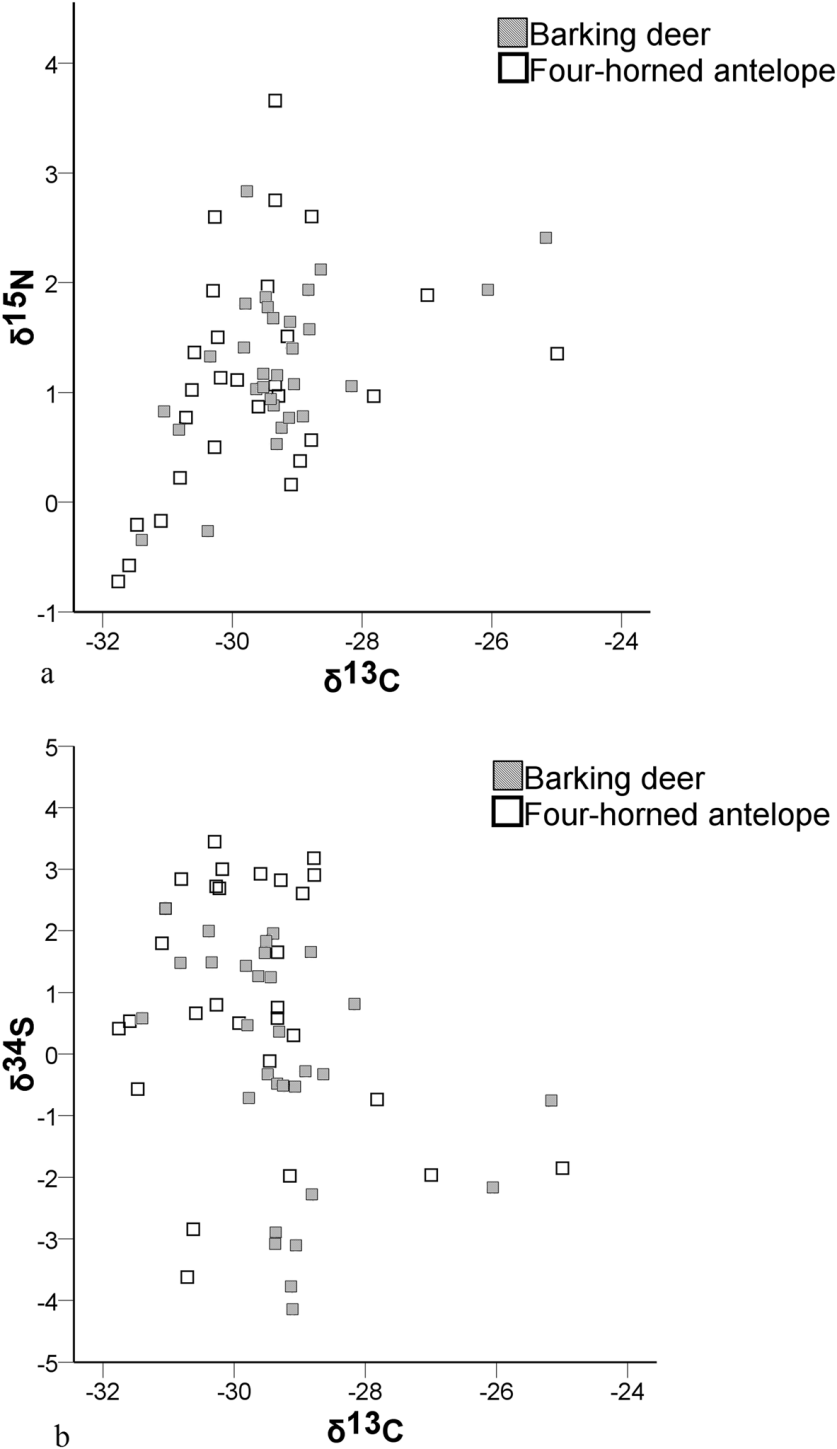
Scatter plots of stable isotopes of faeces of barking deer (n = 30) and four-horned antelope (n = 28). **(a)** δ^13^C and δ^15^N signatures and **(b)** δ^13^C and δ^34^S signatures.

Faecal δ^13^C values for BD (−29.3 ± 1.2) and FHA (−29.7 ± 1.4) were similar. Most published studies used stable C signatures of plant species from the study site to obtain the proportion of C_3_/C_4_ composition from faeces of study animal [15,18,31]. Here, we used the global stable C signatures for plants to compare our findings. We assumed that stable C signatures for the plants in Bardia National Park are similar to the range of stable C signatures for global C_3_/C_4_ plants, i.e., δ^13^C values ranged from −22 to −37‰ with a mean of −27‰ for C_3_ plants, while C_4_ plants ranged from −9‰ to −15‰ with a mean of −12.5‰ [45,46]. Despite the fact that the majority of grass species available to herbivores in the study area are C_4_ plants [47-50], faecal δ^13^C values from our study confirmed that the bulk of the BD and FHA diets consisted of C_3_ plants. Hence, our study confirmed that both species are browsers and their diet is composed of C_3_ plants with consistent nitrogen (δ^15^N and %N) levels. Furthermore, similar δ^13^C values (Figure 1) for the study species indicated their dietary niches completely overlapped along the coarse level of the browser-grazer continuum.

If resources are not limiting population growth, co-existence of several species and the degree to which they overlap in their resource use are not a measure of competition, but are actually indicators of their similarities to one another [51]. Hence, completely overlapping preferences for browsing C_3_ plants over grazing C_4_ grasses signifies the similarities in feeding behaviour of these ungulates in lowland Nepal. Our findings that FHA are browsers are compatible with the findings of Sharma *et al*. [35] and Solanki & Naik [40]. In an experiment in India, though the grass density was higher in experimental plots, Solanki & Naik [43] found that FHA diets contained only about 9.41% grass. Similarly, Sharma *et al*. [38] also confirmed the preference of this species for browsing over grazing in their cafeteria experiment. Furthermore, our findings of BD as browsers are compatible with the findings of Barrette [39] in Sri-Lanka and Teng *et al*. [52] in Hainan Island, China. These authors mentioned this species as a browser with preference for forbs, fruits and young shoots rather than grass. But our findings contradict the findings of Yonzon [41], who mentioned BD as grazers in Chitwan National Park, Nepal and of Nagarkoti and Thapa [42] as a mixed feeder in the mid-hill region of Nepal. Such a difference in diet of the BD might be because of their higher adaptability to different habitat conditions. BD are widely distributed from south Asia to south-east Asia and from lowland to the high mountains [53], whereas the FHA is an endemic species with a narrow range of distribution only in the lowlands of the Indian sub-continent [38,54]. Moreover, BD use a variety of habitats, from dense forest in south-Asia [39,55] to scrub grassland and thorny shrub land in Hainan Island, China [52], whereas FHA inhabit relatively open and dry forest in hilly terrain [38,56]. BD exhibit no seasonal home range [37]. Therefore, unlike FHA, it appears that BD have a greater adaptability to the habitat conditions and resource availability [57], leading to a wider range of feeding habits.

Another important finding of this study, which cannot be covered by microscopic techniques of dietary analysis [58] alone, is higher faecal δ^34^S values for FHA than for BD. Such differences in δ^34^S values are perhaps due to the different foraging habitats of these animals because δ^34^S values of plants are regulated by the δ^34^S values of underlying local bedrock and microbial activities in soils [59,60]. Furthermore, in comparison to BD, FHA were more frequently encountered at mineral lick sites in Babai valley (personal observation). Perhaps the BD and FHA have differential preferences for the minerals that also contribute to the high variability in faecal δ^34^S values.

### Intraspecific variation in seasonal diets

**Figure 2:**
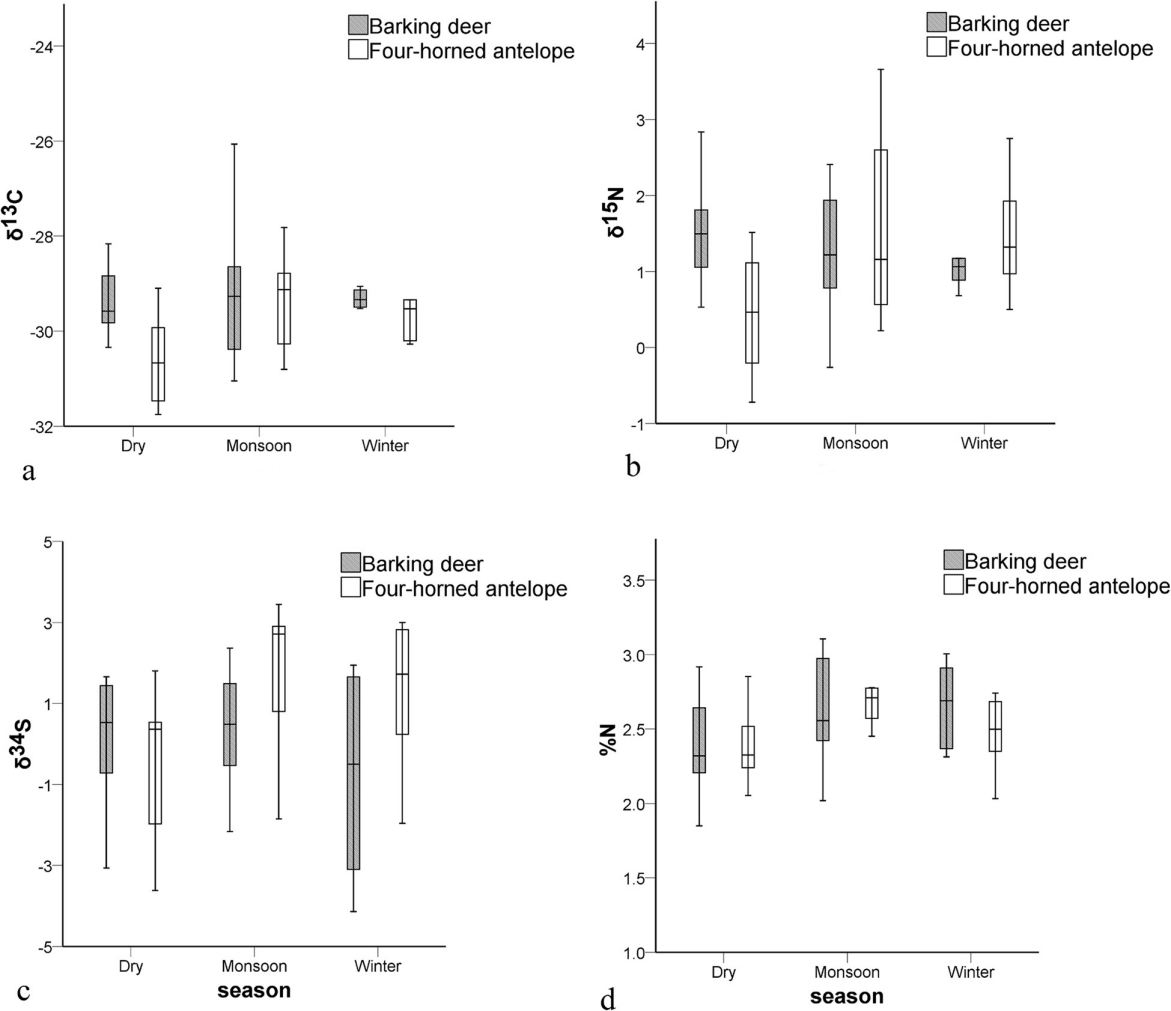
Box plots showing seasonal variation in faecal stable isotopes of barking deer and four-horned antelope. **(a)** δ^13^C, **(b)** δ^15^N, **(c)** δ^34^S, and **(d)** %N values for the study species in lowland Nepal, with the interspecific as well as intraspecific dietary variation.

Our analyses revealed that there was no significant seasonal effect on faecal stable isotopes of BD (MANOVA; Pillai’s Trace: F_8,50_ = 1.926, *p* = 0.077). However, there was a significant seasonal effect on FHA isotope values (MANOVA; Pillai’s Trace: F_8,46_ = 3.528, *p* = 0.003). One-way ANOVA revealed the difference in faecal δ^13^C: F_2,25_ = 4.52, *p* = 0.021, R^2^ = 0.27; δ^15^N: F_2,25_ = 4.29, *p* = 0.025, R^2^ = 0.26; and δ^34^S: F_2,25_ = 4.35, *p* = 0.024, R^2^ = 0.26 (Figure 2, Additional file 1). Post-hoc analysis of FHA isotope values indicated that mean isotope values obtained during the dry season were significantly lower than that of the monsoon season for δ^13^C (*p* = 0.019), δ^15^N (*p* = 0.036), and δ^34^S (*p* = 0.025). Winter season isotope values of the FHA were similar to values obtained during the dry (δ^13^C: *p* = 0.145, δ^15^N: *p* = 0.067, and δ^34^S: *p* = 0.100) and monsoon (δ^13^C: *p* = 0.690, δ^15^N: *p* = 0.980, and δ^34^S: *p* = 0.860) seasons (Figure 2). Results of the ANOVA showed no significant season effect on BD or FHA faecal %N (BD: F_2,27_ = 2.51, *p* = 0.100, R^2^ = 0.16; FHA: F_2,25_ = 1.43, *p* = 0.250, R^2^ = 0.10) (Figure 2).

Stable isotope ratios differed significantly for FHA throughout the seasons but were consistent for BD (Figure 2). The main habitat of BD, i.e., the riverine forest and Sal forests of Terai, is less susceptible to wildfire and water scarcity than the preferred habitat of FHA, i.e., hill sal forest and deciduous hill forest. Since water stress and forest fires affect the physiology of plants, and hence, the stable isotopes of the vegetation community [61-63], feeding on the same plant species from the same habitat types with consistent plant δ^13^C and δ^15^N signatures is possible for BD but not for FHA. Furthermore, due to limited resources, FHA might have changed their feeding strategies by 1) shifting their diet to other parts of the same plant species that either remain unaffected by fire or are still edible after fire, e.g., bark; and/or 2) expanding their home range to another forest type to fulfil their energy requirements, foraging for the same plant species. Such a shift in diet without compromising diet quality might have led to the seasonal variation in δ^13^C, δ^15^N and δ^34^S values for FHA.

### Interspecific variation in seasonal diets

From the comparison of faecal stable isotope ratios between different seasons, we found that δ^13^C (F = 6.238, *p* = 0.022) and δ^15^N (F = 6.478, *p* = 0.020) values were significantly higher in the dry season diet of BD than that of FHA; δ^34^S values (F = 5.188, *p* = 0.037) were significantly lower in the winter diet of BD than that of FHA, whereas isotope signatures were similar in the monsoon diets of both species. Diet quality in terms of %N was consistent throughout the seasons (Additional file 1 and Figure 2).

Faecal stable isotope ratios confirmed the seasonal niche portioning between BD and FHA for the dry season along the δ^13^C and δ^15^N axis and along δ^34^S axis for the winter season, but faecal stable isotope ratios were consistent for the monsoon season (Additional file 1 and Figure 2). These differences may correspond to the plant available moisture and availability of resources because plant available moisture, which affects δ^13^C as well as δ^15^N values of plants [60], varies in different habitats even within the same season, leading to the different isotopic signatures for the same plant species. Furthermore, the monsoon season is the resource-rich period, with ample water resources and soft ground vegetation; but with the onset of winter, air moisture levels decrease and seasonal streams dry up. Dry season forest fires further increase resource scarcity. Furthermore, the quality of available diet in the dry season is poor [64] because most plants in lowland Nepal start to sprout with the pre-monsoon rains at the end of the dry season [65,66].

In a study that applied faecal pellet belt transect surveys during the dry season in the same study site, Pokharel *et al*. [57] found strong evidence that FHA preferred hill sal forest and deciduous hill forest at higher elevations, whereas BD preferred riverine and sal forest at lower elevations. This is complementary to our faecal isotopic data obtained from the dry season for the two ungulates. Therefore, we suggest that the diets of BD and FHA are likely to be composed of different plant species. However, it is yet to be explored if the plants were the same species but of different habitat origin. Despite the interspecific variations in faecal δ^13^C, δ^15^N, and δ^34^S values for different seasons, the diet quality in terms of %N was consistent for both species (Additional file 1 and Figure 2). This indicates that BD and FHA are both capable of partitioning the resources under resource-limited conditions and fulfilling their energy requirements without compromising diet quality. Our study furthermore supports theoretical expectations that sympatric animals should reduce competition by filling different trophic niches, that diet overlap should be greater among similar sized animals, and that diet overlap should decrease with decreasing food resources [30]. Similar to the findings of Dunbar [67] on high altitude herbivores in Ethiopia, and Prins *et al*. [68] on bovid species in southern Mozambique, our results support the hypothesis that dietary overlap decreases during the dry season when available food is in short supply. Hence, our prediction that BD and FHA depend on different dietary sources is partially supported. According to our expectations, we found more pronounced differences in dietary sources (as reflected by faecal isotopic values) during seasons when resources were limited. Indeed, an earlier study by Pokharel *et al*. [57] emphasized that differential resource use on hill sal and deciduous hill forest by FHA, particularly during the dry season, facilitated the niche differentiation that allowed species to co-exist. We hypothesize for future investigations that FHA use different habitats in different seasons and migrate seasonally at the local level, while BD do not.

## Conclusion

Barking deer and four-horned antelope diet is composed of C_3_ plants, hence they are browsers. They have overlapping trophic niches in browser/grazer continuum relative to δ^13^C, but have partitioned their trophic niche along the δ^34^S axis. On a seasonal scale, we found completely overlapped trophic niches during monsoon season, but the degree of resource partitioning increased during winter and peaked during the dry season. Intraspecific seasonal diet was consistent for barking deer throughout the seasons, but varied for four-horned antelope. Such seasonal variability and resource partitioning, explained by faecal δ^13^C, δ^15^N and δ^34^S values, was possible mainly because of the dietary shift of four-horned antelope. We hypothesize for future research that four-horned antelope is a weaker competitor and exhibit seasonal migration at the local level to solve the seasonal variability problem. In Bardia National Park and other parts of the lowland Nepal, focusing on dry season resource management to reduce the potential competition for limited resources is likely to be most successful, particularly for four-horned antelope.

## Methods

### Study area

**Figure 3:**
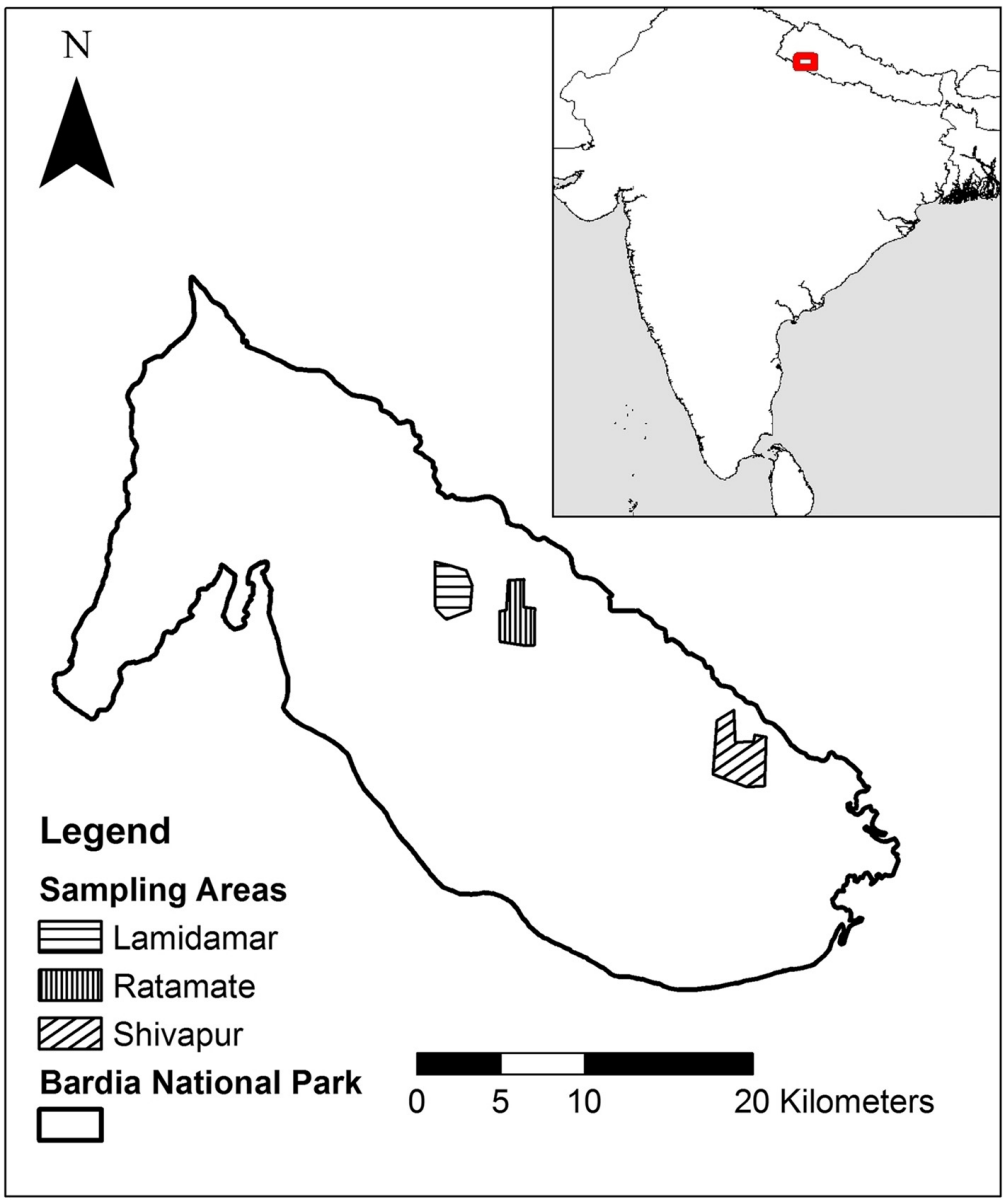
Survey areas within the Babai valley, Bardia National Park, Nepal. Their delineation encompasses the outermost sampling points.

We conducted this study in Babai valley in the south-eastern part of Bardia National Park (28^o^ 23′ 0″ N, 81^o^ 30′ 0″ E) in Nepal. The park is located in the Terai, the lowlands near the Indian border 390 km west of Kathmandu (Figure 3). It is the largest national park in the Terai covering an area of 968 km^2^ (www.dnpwc.gov.np assessed on 19 March 2014). The park has a subtropical monsoonal climate with three distinct seasons: monsoon (June to September), winter (October to February) and dry (March to May) seasons. Monthly mean temperature of the area ranges from a minimum of 10°C in January to a maximum of 45°C in June. Most of the rainfall occurs during the monsoon season (1560–2230 mm) from June to September (Department of Hydrology and Meteorology, Nepal: 2004 to 2009 unpublished data). The vegetation within the study area is sub-tropical, consisting of a mosaic of floodplain communities with riverine forest and climax sal *Shorea robusta* forest with patches of grassland (locally known as *phanta*). Tree species composing the upper canopy include *Shorea robusta*, *Terminalia tomentosa*, *Mallotus philippensis*, *Acacia catechu*, *Dalbergia sissoo*, *Schleicheria trijuga, Pinus roxburghii*, *Buchanania latifolia*, and *Bombax ceiba* while forest understory and grassland are dominated by grass species such as *Saccharum spontaneum, S. ravennae, Vitiveria zizanoides, Imperata cylindrica, Cynodon dactylon, Erianthus ravennae, Eulaliopsis binata* and *Desmostachia bipinnata* [33,69]. Most of the tree and shrub species found in the area are C_3_ while the grass species are C_4_ plants [47-50].

### Sample collection and stable isotope analysis

Fresh faecal samples were collected from three different parts [i.e., Lamidamar, Ratamate (monsoon samples) and Shivapur (*ca*. 250–800 m a.s.l.) of the study area (Figure 3)]. Faecal samples were identified as ‘fresh’ if they were less than two days old, which was visually assessed based on the colour, texture and smell. We considered only those faecal pellets for collection, which were not contaminated by fungi, soil and insects. At least 18 samples were collected, and each sample was from a different dung pile of each species for each season (Additional file 2). Samples were first air-dried in the field, and then oven dried at 60°C for 24 hrs in the lab, thereafter mill-grounded through a 1 mm sieve into a homogenous powder.

Approximately 1.55 mg of sample was weighed in a small tin cup using a micro-analytical balance. Samples were combusted using the vario Micro cube elemental analyzer (Elementar, Analysensysteme GmbH, Germany) and the resultant CO_2_, N_2_ and SO_2_ gases were introduced into a Micromass Isoprime isotope ratio mass spectrometer (Isoprime Ltd., Cheadle Hulme, UK) via a continuous flow-through inlet system. Sample ^13^C/^12^C, ^15^ N/^14^ N and ^34^S/^32^S ratios are expressed in the delta (δ^13^C, δ^15^N and δ^34^S) notation in parts per million (‰). Those values are relative to the following standards: the Vienna Pee Dee Belemnite (VPDB) for carbon, atmospheric N_2_ for nitrogen, and sulphanilamide-calibrated and traceable to NBS-127 (barium sulphate, δ^34^S = +20.3‰) for sulphur. We obtained stable isotope ratios by using the equation:

$$ \delta X=\left[\left(\frac{R_{sample}}{R_{standard}}-1\right)\right]\times 1000 $$

where *X* is ^13^C or ^15^ N or ^34^S and *R* is ^13^C/^12^C or ^15^ N/^14^ N or ^34^S/^33^S. Internal laboratory standards indicate that our measurement errors (SD) were ± 0.15%, 0.05% and 0.05% for δ^15^N, δ^13^C, and δ^34^S, respectively.

### Statistical analysis

For each species and element tested separately, q-q plots showed that variables were normally distributed. A Multivariate Analysis of Variance (MANOVA) [70] was applied where δ^13^C, δ^15^N, δ^34^S, and %N were used as dependent variables; and species, sampling sites and seasons as main effects. Only seasons and interaction between species and season (species × seasons) had a significant effect on dependent variables (Table 1). Therefore, we decided to omit the main effect variable ‘sampling sites’ from further analysis. After the MANOVA was conducted with season as a main effect variable, an one-way analysis of variance (ANOVA) followed by a Tukey’s HSD post-hoc test [70] was performed for each dependent variable to detect differences between seasons for individual species (intra-specific seasonal variation). Seasons were analysed separately with species as a main effect variable for interspecific seasonal variation. We used IBM SPSS statistics version 20 (IBM corporation 2011, Armonk, NY, USA) for all statistical analysis and developing graphs.

**Table 1:** **MANOVA (Pilli-test) results for stable isotopes of faeces of barking deer and four-horned antelope**

**Variables**	**df**	**F**	***p***
Area	2	0.474	0.872
Species × Area	2	1.353	0.227
Species	1	1.475	0.224
Season	2	3.704	0.001
Species × Season	2	2.611	0.012

Statistics include the degrees of freedom (df), F-ratio (F) and their significance level (*p*) to show the variations in a combination of stable isotopes of faeces (δ^13^C, δ^15^N, and δ^34^S signatures, and %N) of the study species for study sites (area), season and their combinations.

## Additional files

Additional file 1:
**Results from an ANOVA for stable isotopes of faeces of barking deer and four-horned antelope.** Statistics include degrees of freedom (df), mean ± standard deviation (SD), F-ratio (F), their significance level (*p*) and variances explained (R^2^) to show the interspecific variations in diets of the study species for dry, monsoon and winter seasons. Additional file 2:
**Season, sites and dates of faecal sample collection of barking deer and four-horned antelope.**

## Abbreviations

BD: Barking deer
FHA: Four-horned antelope
MANOVA: Multivariate analysis of variance
ANOVA: Analysis of variance
