# Supplementary material for: Isotopic evidence for dietary niche overlap between barking deer and four-horned antelope in Nepal
Source: J Biol Res (Thessalon). 2015 May 6;22(1):6. doi: 10.1186/s40709-015-0029-0 (PMC4440280; doi:10.1186/s40709-015-0029-0)
Supplement: Additional file 1: — Results from an ANOVA for stable isotopes of faeces of barking deer and four-horned antelope. Statistics include degrees of freedom (df), mean ± standard deviation (SD), F-ratio (F), their significance level (p) and variances explained (R2) to show the interspecific variations in diets of the study species for dry, monsoon and winter seasons. [file 40709_2015_29_MOESM1_ESM.pdf]

Additional file 1. Results from an ANOVA for stable isotopes of faeces of barking deer and four-horned antelope.

Seasons	Variable	df	BD (Mean ± SD)	FHA (Mean ± SD)	F	p	R^2
Dry	$\delta^{13}C$	1, 18	-29.6 ± 0.9	-30.6 ± 0.9	6.23	0.02	0.26
	$\delta^{15}N$	1, 18	1.4 ± 0.8	0.4 ± 0.8	6.48	0.02	0.26
	$\delta^{34}S$	1, 18	0.1 ± 1.6	-0.5 ± 1.7	0.54	0.47	0.29
	%N	1, 18	2.37 ± 0.3	2.4 ± 0.2	0.01	0.92	0.001
Monsoon	$\delta^{13}C$	1, 18	-28.9 ± 1.9	-28.9 ± 1.6	0.00	0.98	0.001
	$\delta^{15}N$	1, 18	1.3 ± 0.8	1.5 ± 1.1	0.33	0.57	0.02
	$\delta^{34}S$	1, 18	0.4 ± 1.4	1.8 ± 1.8	3.32	0.08	0.16
	%N	1, 18	2.6 ± 0.3	2.6 ± 0.4	0.00	0.99	0.00
Winter	$\delta^{13}C$	1, 16	-29.3 ± 0.7	-29.4 ± 1.0	0.11	0.75	0.01
	$\delta^{15}N$	1, 16	1.1 ± 0.4	1.5 ± 0.7	1.66	0.22	0.09
	$\delta^{34}S$	1, 16	-1.0 ± 2.4	1.3 ± 1.8	5.18	0.03	0.25
	%N	1, 16	2.7 ± 0.2	2.5 ± 0.2	2.59	0.13	0.14
All	$\delta^{13}C$	1, 56	-29.3 ±1.2	-29.7±1.4	1.37	0.24	0.19
	$\delta^{15}N$	1, 56	1.2±0.7	1.1 ±1.0	0.31	0.57	0.18

$\delta^{34}S$	1, 56	-0.2±1.8	0.8±2.0	4.60	0.04	0.24
%N	1, 56	2.5±0.3	2.5±0.3	0.68	0.41	0.14

Statistics include degrees of freedom (df), mean ± standard deviation (SD), F-ratio (F), their significance level (p) and variances explained (R^2) to show the interspecific variations in diets of the study species for dry, monsoon and winter seasons.
