# Supplementary material for: Isotopic evidence for dietary niche overlap between barking deer and four-horned antelope in Nepal
Source: J Biol Res (Thessalon). 2015 May 6;22(1):6. doi: 10.1186/s40709-015-0029-0 (PMC4440280; doi:10.1186/s40709-015-0029-0)
Supplement: Additional file 2: — Season, sites and dates of faecal sample collection of barking deer and four-horned antelope. [file 40709_2015_29_MOESM2_ESM.pdf]

Additional file 2: Season, sites and dates of faecal sample collection of barking deer and four-horned antelope.

Season	Sampling sites	Date	BD	FHA	Total
Dry	Lamidamar	Mar-2012	6	3	9
	Ratamate	Apr-2012	4	7	11
Monsoon	Ratamate	Sep-2012	10	10	20
Winter	Shivapur	Dec 2012/January 2013	10	8	18
	Total		30	28	58
